# Terminal Mannose Residues in Seminal Plasma Glycoproteins of Infertile Men Compared to Fertile Donors

**DOI:** 10.3390/ijms160714933

**Published:** 2015-07-02

**Authors:** Beata Olejnik, Anna Jarząb, Ewa M. Kratz, Mariusz Zimmer, Andrzej Gamian, Mirosława Ferens-Sieczkowska

**Affiliations:** 1 Department of Chemistry and Immunochemistry, Wrocław Medical University, Bujwida 44A, 50-345 Wrocław, Poland; E-Mails: olejnik.k.beata@gmail.com (B.O.); ewa.kratz@umed.wroc.pl (E.M.K.); 2 Institute of Immunology and Experimental Therapy, Polish Academy of Science, Rudolfa Weigla 12, 53-114 Wrocław, Poland; E-Mail: anna.jarzab@iitd.pan.wroc.pl; 3 2nd Department and Clinic of Gynaecology, Obstetrics and Neonatology, Wrocław Medical University, Borowska 213, 50-556 Wrocław, Poland; E-Mail: mariusz.zimmer@umed.wroc.pl; 4 Department of Clinical Biochemistry, Wrocław Medical University, Chałubińskiego 10, 50-368 Wrocław, Poland; E-Mail: andrzej.gamian@umed.wroc.pl

**Keywords:** fertility marker, glycosylation, male infertility, high-mannose type glycans, seminal plasma

## Abstract

The impact of seminal plasma components on the fertilization outcomes in humans is still under question. The increasing number of couples facing problems with conception raises the need for predictive biomarkers. Detailed understanding of the molecular mechanisms accompanying fertilization remains another challenge. Carbohydrate–protein recognition may be of key importance in this complex field. In this study, we analyzed the unique glycosylation pattern of seminal plasma proteins, the display of high-mannose and hybrid-type oligosaccharides, by means of their reactivity with mannose-specific *Galanthus nivalis* lectin. Normozoospermic infertile subjects presented decreased amounts of lectin-reactive glycoepitopes compared to fertile donors and infertile patients with abnormal semen parameters. Glycoproteins containing unveiled mannose were isolated in affinity chromatography, and 17 glycoproteins were identified in liquid chromatography-tandem mass spectrometry with electrospray ionization. The *N*-glycome of the isolated glycoproteins was examined in matrix-assisted laser desorption ionization mass spectrometry. Eleven out of 27 identified oligosaccharides expressed terminal mannose residues, responsible for lectin binding. We suggest that lowered content of high-mannose and hybrid type glycans in normozoospermic infertile patients may be associated with impaired sperm protection from preterm capacitation and should be considered in the search for new infertility markers.

## 1. Introduction

Gamete fusion and some regulatory processes in the fertilization cascade require specific carbohydrate structures [1,2,3]. This is not surprising, as sugar epitopes are known to provide a system of biological information transfer, and they are fundamental in mediation of a great number of cellular communication events [4,5]. Interestingly, some individual proteins of seminal plasma, as well as the glycome as a whole, present glycosylation patterns that are rare in human secretions. One of them is the presence of high-mannose and hybrid type oligosaccharides [6,7].

The *N*-glycosylation pathway in higher vertebrates is divided into two parts. At the beginning, 14-monosaccharide glycan with two *N*-acetylglucosamines (GlcNAc), nine mannoses (Man) and three terminal glucoses are synthesized on the membrane-anchored dolichyl phosphate and transferred *en bloc* to the glycosylation sequon in the nascending protein. Next, the oligosaccharide is hydrolyzed to the 5-monosaccharide core that is further rebuilt with complex-type antennae, containing GlcNAc, galactose and sialic acid instead of six Man residues. Glycans of this type are considered as a “passport”, enabling secretion of the glycoprotein outside the cell. Thus, high-mannose type glycans are absent (or detectable only in trace amounts) in human secretory glycoproteins [8].

This unusual type of glycosylation was described as necessary in seminal plasma glycodelin (GdS), the glycoprotein responsible for keeping sperm uncapacitated until it reaches the upper parts of the female reproductive tract [9,10,11]. In the fallopian tubes, GdS is replaced with female isoforms: glycodelins A and F, and the sperm activation process may be initiated. Male and female isoforms of glycodelin differ only in their glycosylation patterns, so the unique oligosaccharide structure of GdS seems to be crucial in the regulation process.

The high-mannose and hybrid type glycans were also reported in two common seminal plasma glycoproteins, prostate specific antigen (PSA) and prostatic acid phosphatase (PAP), in benign prostate hyperplasia and cancer [7,12,13], but so far they have not been analyzed in the context of male fertility.

In recent decades, decreasing male fertility has become a social and medical problem in industrial countries [14,15,16,17,18]. Conception failures are most often associated with poor semen parameters. Nevertheless, conception problems often also affect normozoospermic men. Recently, some andrologists have claimed that standard semen analysis is not a sufficient predictive factor for male subfertility or infertility [14,16,19].

In our earlier studies, we found some alterations in the glycosylation profile in seminal plasma (SP) glycoproteins of men with impaired fertility. These studies, however, were focused on the glycosylation patterns commonly regarded as disease-related, such as sialylation and fucosylation [20,21,22,23].

In this study we aimed to screen SP of subfertile/infertile men for the presence of glycoproteins decorated with glycans containing at least one antenna terminated with unveiled mannose, and compare the profile of this mannose expression to fertile subjects, in order to find possible markers of impaired fertility. Special focus was directed at patients with normal sperm parameters, as prediction of fertilization outcomes in this group is particularly difficult. *Galanthus nivalis* lectin (GNL) was applied to detect oligosaccharides of interest. We also attempted to identify glycoproteins that can carry such a unique glycoepitope.

## 2. Results

### 2.1. Detection of High-Mannose Type Oligosaccharides

**Figure 1:**
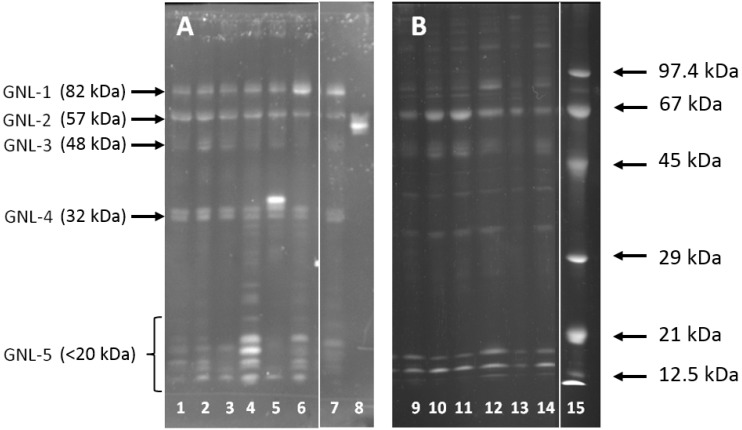
GNL reactivity pattern of seminal plasma glycoproteins. (**A**) Western-blot probed with GNL; (**B**) Sypro Ruby stained gel. Lanes 1–7 and 9–14—individual samples, lane 8—carboxypeptidase Y, lane 15—molecular mass markers.

**Figure 2:**
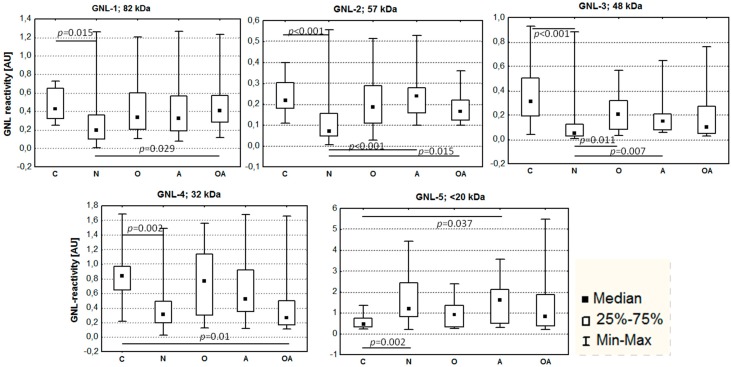
GNL reactivity of seminal plasma glycoproteins. Infertile patients: N—normozoospermia, O—oligozoospermia, A—asthenozoospermia, OA—oligoasthenozoospermia; C—control fertile subjects. *p* values in Kruskal–Wallis test represent statistical significance of the difference among the analyzed groups.

The representative pattern of GNL reactivity in seminal plasma proteins is shown in Figure 1. At least five GNL-reactive bands were detected in all the samples. Four of them were easily accessible for densitometric analysis, and their molecular masses were calculated as follows: GNL-1—82 kDa, GNL-2—57 kDa, GNL-3—48 kDa, GNL-4—32 kDa. In the gel region <20 kDa, 1–10 bands were present in particular samples, and they were summarized as GNL-5 for the densitometric analysis. Total lectin reactivity in the subjects classified in different semen groups according to the results of standard semen analysis is shown in Figure 2. In all four single bands, GNL reactivity of normozoospermic infertile (N) subjects was significantly lower than in the fertile controls (C), with *p* values in the Kruskal–Wallis test varying from 0.015 to less than 0.001. This lowered lectin reactivity was significant also when compared to the oligoasthenozoospermic (OA) group in GNL-1, asthenozoospermic (A) and OA in GNL-2, and oligozoospermic (O) and A in GNL-3. In contrast, total reactivity of the bands <20 kDa (GNL-5) was higher in N than in the control group. A decreased total amount of unveiled mannose was also observed in GNL-4 of the OA group when compared to controls. In the GNL-5 fraction, lectin reactivity was elevated in group A *vs.* controls (Figure 2).

**Figure 3:**
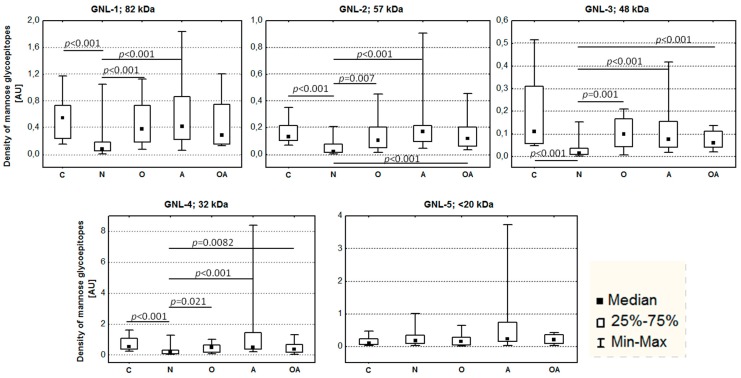
Density of mannose glycoepitopes in seminal plasma glycoproteins. Infertile patients: N—normozoospermia, O—oligozoospermia, A—asthenozoospermia, OA—oligoasthenozoospermia; C—control fertile subjects. *p* values in Kruskal–Wallis test represent statistical significance of the difference among the analyzed groups.

A highly significant difference of normozoospermic fertile and infertile subjects was confirmed also when the density of mannose glycoepitopes was estimated as the ratio of total lectin reactivity to protein content in bands GNL-1–4 (*p* < 0.001, Figure 3). The decrease of GNL-reactive glycoepitopes in N subjects was also profound when compared to the other groups, *i.e.*, O, A and OA, in bands GNL-1–4. In GNL-5, there were no statistically significant differences among the analyzed groups.

### 2.2. Altered Electrophoretic Patterns of GNL Reactivity

**Figure 4:**
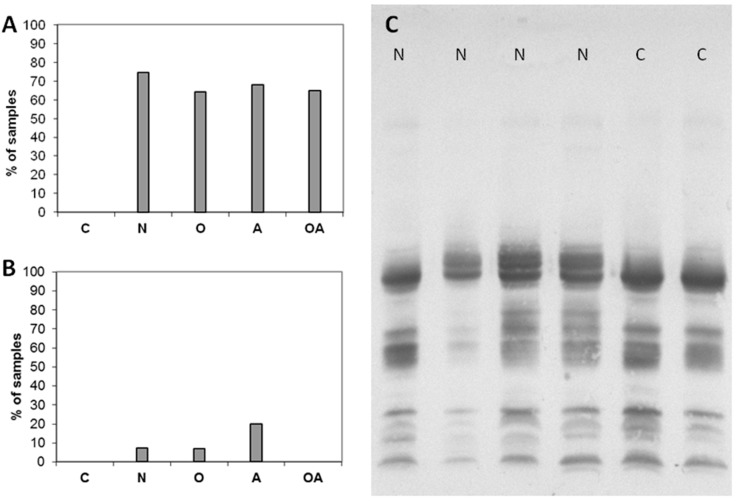
Altered patterns of GNL-reactive glycoproteins. (**A**) Percentage of samples with increased number of GNL-reactive bands <20 kDa; (**B**) Percentage of samples with abundant GNL-4 band of increased mass in PSA band; (**C**) Reactivity of seminal plasma samples with anti-PSA antibodies. N—normozoospermia, O—oligozoospermia, A—asthenozoospermia, OA–oligoasthenozoospermia, C–control fertile subjects.

In some of the analyzed samples, the number of GNL-reactive bands in the gel region < 20 kDa was especially high (Figure 1A, lanes 4 and 6). In Figure 4A, the percentage of samples with at least seven GNL-reactive bands <20 kDa is shown. These low molecular mass GNL-reactive bands were completely absent in normozoospermic fertile subjects, but were almost equally distributed within all the infertile groups.

Another atypical pattern was the presence of an additional intensive band with higher mass in the region of GNL-4 (Figure 1A, lane 5). This band was also absent in fertile controls and present in 10%–20% of samples in the infertile N, O and A groups (Figure 4B).

In our previous studies, we determined the reactivity with anti-PSA antibodies in the above-mentioned gel regions [23]. The samples exhibiting an unusual GNL-reactivity pattern were probed with anti-PSA antibodies (Figure 4C) and the content of PSA fragments in these bands was confirmed.

Fragmentation of seminal plasma proteins results from proteinase digestion during liquefaction of semen coagulum. As our results indicate, apart from fibronectin and semenogelin, it also includes PSA autolysis.

### 2.3. Identification of GNL-Reactive Bands in Mass Spectrometry

GNL-reactive glycoproteins representing bands 1–4 were excised from the gel after electrophoresis and submitted to mass spectrometric identification of proteins. Instead of the wide gel region of GNL-5, the glycoprotein band of weak GNL reactivity and molar mass corresponding to glycodelin S was included in the analysis as GNL-6. As is usual in 1D SDS-PAGE, numerous proteins were present in each single band. In Table 1, only the matches with a score number >500 are shown. Albumin, keratin contaminants and unknown/unidentified proteins were excluded.

**Table 1:** Mass spectrometry identification of glycoproteins isolated from SDS-PAGE gels.

Band No.	Protein Results of Mascot Database Search	Score	Matches	Antibody Reactivity [23]
GNL-1	Chain X, structure of recombinant human lactoferrin	6473	148	Fn, PSA, Tf
	lactoferrin precursor	6405	148	
	lactoferrin	6379	146	
	fibronectin precursor	6334	145	
	lactoferrin	2004	41	
	fibronectin 1, isoform CRA_f	1517	29	
	serotransferrin precursor	872	25	
	PREDICTED: mucin-6 isoform	716	15	
	extracellular matrix protein 1	508	12	
GNL-2	PRO2044	3056	78	Fn, hCG, ATIII
	galectin-3-binding protein precursor	1716	32	
	fibronectin isoform 3 preproprotein	1705	35	
	lactoferrin	1666	37	
	prosaposin isoform a preproprotein	1071	25	
	serotransferrin precursor	975	24	
	l-plastin variant	929	22	
	heat shock protein	656	12	
GNL-3	prostatic acid phosphatase isoform PAP precursor	3516	98	PAP, IgG
	fibronectin precursor	2420	51	
	Chain X, Structure Of Recombinant Human Lactoferrin	1982	40	
	plasma serine protease inhibitor precursor	1723	46	
	Chain A, 2.0 Angstrom Structure Of Intact Alpha-1-Antitrypsin: A Canonical Template For Active Serpins	1341	35	
	Chain A, The S Variant Of Human Alpha1-antitrypsin	1323	36	
	Chain A, Crystal Structure Of Cleaved Antitrypsin Polymer	1285	35	
	Serpin peptidase inhibitor, clade A (alpha-1 antiproteinase, antitrypsin)	1269	34	
	nucleobindin-2 precursor	1066	23	
	brain acid soluble protein 1	977	12	
	2-phosphopyruvate-hydratase alpha-enolase	697	15	
	monocyte differentiation antigen CD14 precursor	642	10	
	extracellular matrix protein 1	621	15	
	serotransferrin precursor	542	14	
	Ig G1 H	517	11	
	Zn-alpha2-glycoprotein	509	13	
GNL-4	prostate specific antigen precursor, partial [Homo sapiens]	3204	64	PSA, PAP
	apolipoprotein J precursor [Homo sapiens]	1662	40	
	fibronectin isoform 3 preproprotein [Homo sapiens]	1198	28	
	Chain A, Human Annexin V With Proline Substitution By Thioproline	893	20	
	annexin A1 [Homo sapiens]	875	18	
	cerebroside sulfate activator protein [Homo sapiens]	659	13	
	acidic epididymal glycoprotein homolog [Homo sapiens]	521	8	
	semenogelin-2 precursor [Homo sapiens]	520	11	
	annexin A3 [Homo sapiens]	488	9	
	semenogelin-1 preproprotein [Homo sapiens]	418	11	
	Zn-alpha2-glycoprotein [Homo sapiens]	391	9	
GNL-6	fibronectin isoform 3 preproprotein [Homo sapiens]	1213	28	Fn, IgG, PSA, GdS
	placental protein 14 [Homo sapiens] *	1207	23	
	semenogelin-1 preproprotein [Homo sapiens]	752	17	
	apolipoprotein J precursor [Homo sapiens]	683	15	
	prostate specific antigen precursor, partial [Homo sapiens]	662	13	
	mucin-6 precursor [Homo sapiens]	616	12	
	protein DJ-1 [Homo sapiens]	581	12	

* glycodelin.

### 2.4. Isolation of GNL-Reactive Glycoproteins

**Figure 5:**
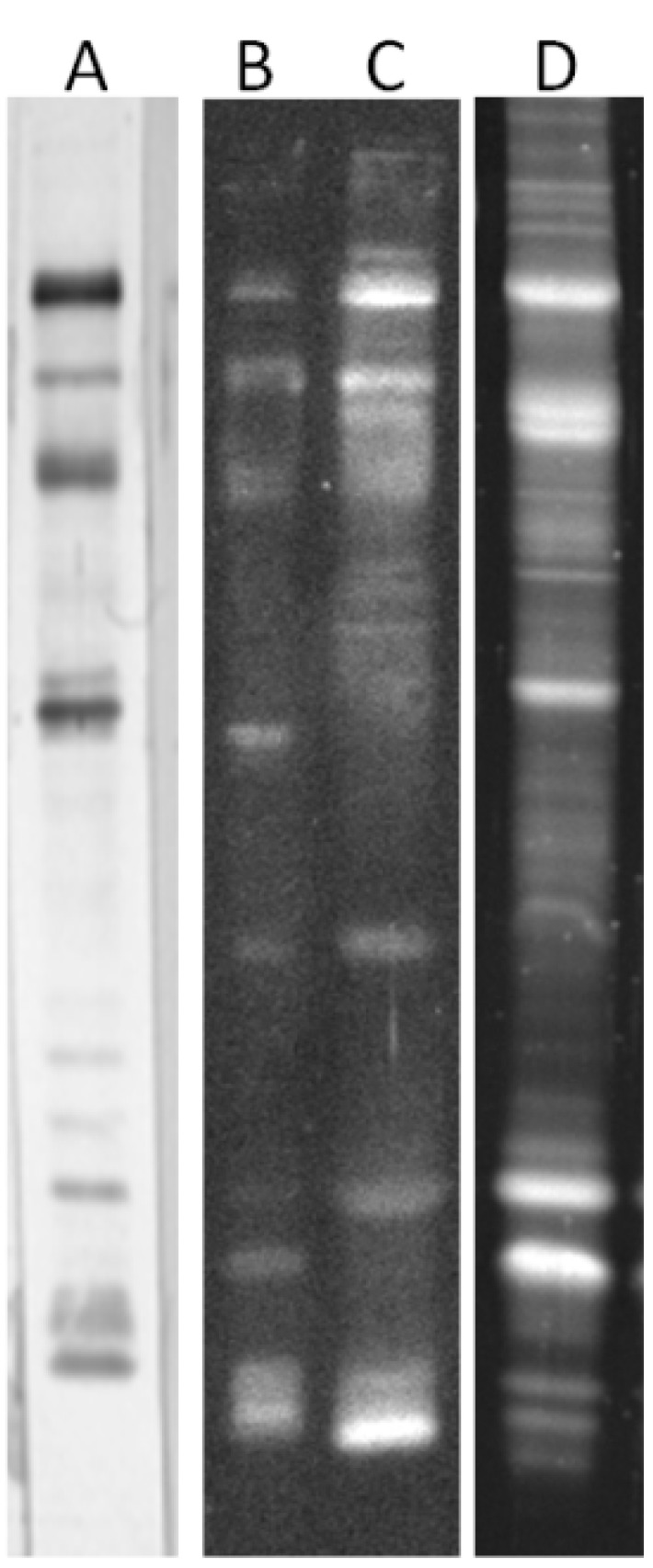
Isolation of GNL-reactive glycoproteins by means of affinity chromatography. (**A**) GNL reactivity of the pooled sample submitted to the column, BCIP/NBT stained; (**B**) GNL reactivity of column unbound glycoproteins, DuoLux stained; (**C**) GNL reactivity of α-methyl-d-mannopyranoside released glycoproteins, DuoLux stained; (**D**) Sypro Ruby stained proteins of α-methyl-d-mannopyranoside released glycoproteins.

To find out which of the glycoproteins identified in SDS-PAGE GNL reactive gel bands really bear a mannose-presenting glycoepitope, we used affinity chromatography to isolate the lectin reactive fraction of SP. Five mg of bulk SP protein pooled from the samples of infertile men was bound to GNL-agarose at pH = 6.5. After washing out the unbound material, lectin-reactive glycoproteins were released from the column with 0.5 M methyl α-d-mannopyranoside in 0.1 M acetate buffer, pH = 4.0. SDS-PAGE and lectin reactivity patterns of bulk material prepared for the chromatography and GNL isolated glycoproteins are shown in Figure 5.

**Table 2:** Mass spectrometry identification of proteins isolated in GNL-chromatography.

Protein Results of Mascot Database Search	Score	Matches
fibronectin	72,742	1563
prolactin-inducible protein	65,038	1179
prostatic acid phosphatase	26,608	682
semenogelin-1	15,725	442
semenogelin-2	14,489	442
clusterin	10,417	336
glycodelin	7447	163
carboxypeptidase E	3190	102
cystatin-S	2136	50
mucin-6	2056	93
lactotransferrin	1923	42
IgGFc-binding protein	1114	32
aminopeptidase N	1015	26
zinc-α_2_-glycoprotein	991	23
calcium-binding protein	499	12
α_1_-acid glycoprotein	444	9
prostate specific antigen	439	11

In the unbound fraction, only weak GNL reactivity was observed. When we compared the column isolated fraction with the crude material in SDS-PAGE, we observed more bands in the GNL-enriched fraction. Those bands included GdS, which was not detectable in the crude material. This protein pattern shows that numerous seminal plasma proteins can carry minor amounts of unveiled mannose responsible for glycan-lectin binding. Thus, the truncation of glycans that leaves mannose accessible for potential ligands seems not to be rare in SP glycoproteins. The results of the Mascot database search after LC–MS/MS analysis are shown in Table 2. The most effective carriers of oligosaccharides capable of binding GNL were identified as fibronectin, prolactin inducible protein (PIP), PAP, semenogelin 1 and 2 and clusterin, detected with score values >10,000 and 330–1500 matched peptide sequences. Lower but still >400 score values were found for the next 11 proteins, including glycodelin, lactotransferrin, Zn α_2_-glycoprotein and PSA.

**Figure 6:**
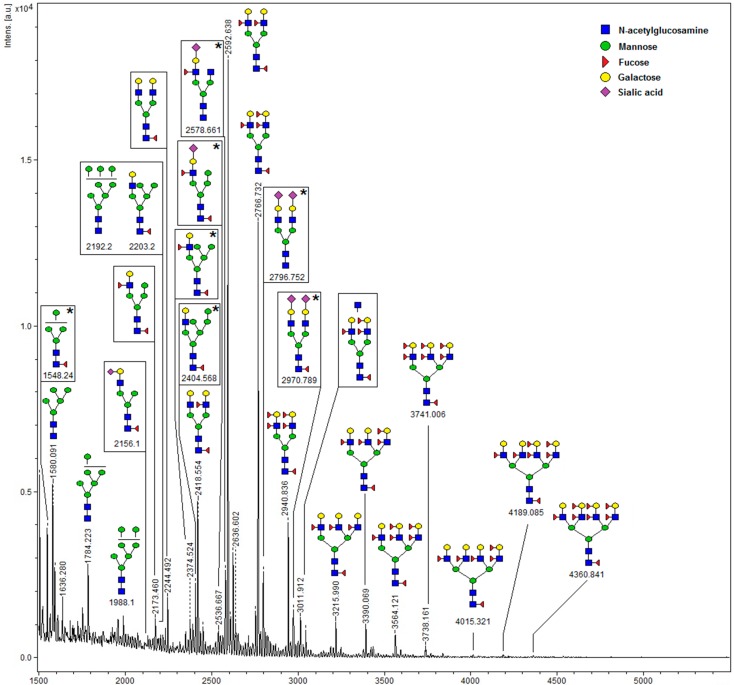
MALDI-TOF-MS profile of oligosaccharides isolated from GNL-reactive seminal plasma glycoproteins. * Structures not reported in [28].

Oligosaccharides from GNL-isolated glycoproteins were released with PNGase F digestion, purified on a C18 column, permethylated and submitted to MALDI-TOF-MS analysis. The spectrum of oligosaccharides with matched glycan structures is presented in Figure 6. Over 20 oligosaccharide structures were identified in the *m*/*z* range from 1550–4361, assuming complete permethylation and single Na^+^ ionization, using GlycoWorkbench software [24,25] and further verified comparing the *m*/*z* values with those presented earlier for the seminal plasma glycome by Pang *et al.* [6]. Dominant glycans were the biantennary complex type, core and antennary fucosylated, and lacking sialic acid. In the *m*/*z* range from 1550–2203, 11 glycans demonstrated unveiled mannose residues, and thus were potential ligands of GNL. Highly branched and overfucosylated oligosaccharides, also reported earlier in the work of Pang *et al.* [6], were present in the GNL-isolated glycoprotein fraction only in minor amounts. Prominent amounts of glycans that cannot bind to lectin indicate that glycoproteins carrying more than one oligosaccharide chain present a glycosylation profile similar to GdS, with at least one glycan of high-mannose, and at least one of complex type.

## 3. Discussion

The impact of SP proteins on the final success or failure of conception is still under discussion [26,27,28]. Some authors claim that the period of sperm–seminal plasma interaction is too short to be decisive in the regulation of fertility, as in humans the ejaculate immediately coagulates to form a vaginal plug and seminal plasma progresses no further than the external cervix [28]. On the other hand, numerous experimental data concerning the fertility of domestic and husbandry animals suggest that this interaction, even when time-limited, is helpful in pregnancy outcomes. Rickard *et al.* [29] showed that seminal plasma improved the ability of epididymal rat sperm to penetrate the cervical mucus. The protective effect of seminal plasma on cryopreserved or sex-sorted spermatozoa was also reported [27,30,31]. Absorption of seminal components may have a positive impact on implantation and subsequent embryo development [28]. The molecular mechanism of these effects is still unknown, though it has been assigned to SP proteins [32,33]. In such a case, the possible role of carbohydrate–protein recognition in this cross-talk should not be neglected.

A few years ago, the seminal plasma glycome was analyzed in several samples of fertile men [6]. Among more than 70 glycans identified, some patterns may be considered as typical for this particular secretion. These are the lack of sialic acid in most glycans, extremely high fucosylation and a significant number of oligosaccharides containing high-mannose type antenna or at least unveiled core mannose. All these features are rare in the other human body fluids [34].

High-mannose type oligosaccharides have been reported in seminal plasma glycodelin (GdS) as necessary and site specific, located at Asn 28. What is important, they are absent in the female isoforms of this glycoprotein. These unique glycans seem to be involved in time and site control of sperm activation [2,9]. The conspicuous content of high-mannose type glycans was also reported in PSA and PAP [7].

After detailed analysis of fucose expression in SP of subfertile men [23], we aimed to study mannose-presenting oligosaccharides in the same set of subjects. In contrast to fucose-displaying glycoepitopes, abundant in over 20 glycoproteins, prominent GNL-reactivity was observed only in a small number of glycoproteins. The four glycoprotein bands pronounced enough for densitometric analysis were co-reactive with fucose-specific *Aleuria aurantia* lectin previously suggested to contain transferrin, PSA, hCG, α_1_-antitrypsin, PAP and IgG [23]. It is possible therefore that different glycoforms of the mentioned glycoproteins are present in SP. The most abundant seminal plasma proteins, *i.e.*, PSA, PAP, fibronectin, semenogelin, PIP and GdS, were all identified as carriers of the GNL-reactive glycoepitope. In their MALDI-TOF-MS glycan profile, 11 oligosaccharides expressed either high-mannose type antenna or unveiled core mannose. Apart from them, prominent amounts of glycans that cannot bind to lectin were present in the sample. This means that in glycoproteins carrying more than one oligosaccharide chain, both types of glycans accompany each other, resembling the glycosylation pattern reported for GdS. In general, the glycan profile shown in this study largely matches that described in the seminal plasma glycome by Pang *et al.* [6], and the difference seems to be quantitative rather than qualitative.

Statistically significant alterations of the amount of GNL-reactive glycoepitopes were observed mainly in the group of normozoospermic infertile patients. In this group, the conception problems do not suggest impaired spermiogenesis, as the number, motility and morphology of sperm are correct, within the WHO normal range. However, as this group of patients is surprisingly large, it is important to determine the causes of conception failure. The results of the current study suggest that searching within the mechanisms of protein–carbohydrate interactions may be fruitful.

To understand the possible role of high-mannose type glycans, investigating the sperm–GdS interaction seems to be crucial. In 2005, Chiu *et al.* [35] reported the presence of two different receptors for GdS on the sperm surface. The carbohydrate moiety of GdS is necessary for binding, as deglycosylation abolishes interaction. Also, female glycoforms of Gd that express a different glycosylation pattern do not act as competitive inhibitors. The authors also demonstrated that GdS–sperm interaction plays an important role as a factor protecting the sperm from capacitation until it traverses the cervical mucus. It is not known which of the two site-specific glycans of GdS is crucial for its binding to the receptor. One of them is the most common, dominant in the whole glycome, asialylated complex structure, and the other one is necessarily a unique high-mannose type. Thus, the expression of high-mannose type glycans may be taken into consideration as an element of the incapacitation mechanism. The decrease of the amount and density of high-mannose type glycans in SP proteins may reflect an impairment of the glycosylation or secretion pathway in seminal vesicles and/or the other secretory glands and cells of the male reproductive tract. As a result, falsely glycosylated GdS cannot saturate sperm surface receptors, failing in its task of maintaining sperm uncapacitated. Next, preterm capacitation weakens sperm activity to fertilize the egg.

Another question worth consideration is whether other glycoproteins presenting a glycosylation profile similar to GdS, as reported in the current study, may also participate in sperm protection against capacitation. Chiu *et al.* described the GdS–sperm interaction as specific, although of low affinity. These authors however tested only related proteins, such as female glycoforms of glycodelin expressing different glycosylation patterns, or other lipocalins, as competitive inhibitors of the interaction. Thus, involvement of other carriers of the unique set of glycans cannot be definitely excluded, especially when the reported low affinity of interaction [35] is taken into account.

To sum up, the content of glycans presenting unveiled mannose in seminal plasma glycoproteins seems to be associated with impaired fertility, even when such impairment is not apparent in the sperm parameters. Some protein candidates should be analyzed in detail to confirm this hypothesis, including PAP, PIP and PSA. GdS seems to be another obvious candidate, but its lower abundance in the seminal plasma may hinder its possible application as a potential biomarker.

## 4. Experimental Section

### 4.1. Clinical Material

Patients enrolled in the study attended the 2nd Clinic of Gynecology and Obstetrics, Wrocław Medical University, for artificial insemination (AI). These were men in the age range 22–56 years, living in childless couples with at least one year of unprotected intercourse without achieving pregnancy. For the participants of the study, the female infertility factor was excluded: all the female partners had normal ovulation and correct ultrasound-checked structure of reproductive organs.

Semen samples were obtained by masturbation after 2–5 days of sexual abstinence, liquefied for one hour at 37 °C, supplemented with an equal volume of Earl’s buffered solution and gently centrifuged (400× *g*, 10 min at room temperature). Spermatozoa were then collected for the insemination procedure, and the spare fraction of seminal plasma, diluted 1:1 with Earl’s solution, was collected for the current study, aliquoted and stored at −80 °C until examined. One hundred and thirty-nine patients were classified in the following groups according to WHO-approved [36] standard semen examination: normozoospermic (N, *n* = 67), oligozoospermic (O, *n* = 14), asthenozoospermic (A, *n* = 25) and oligoasthenozoospermic (OA, *n* = 20).

The control group consisted of 12 healthy fertile volunteers (at least one child fathered), aged 28–38 years. All these subjects were normozoospermic according to WHO standards.

Informed consent of all study participants has been obtained, as well as the approval of the Wrocław Medical University Bioethics Committee (appr. no. KB-631/2012; 25-07-2012).

### 4.2. SDS-PAGE and Lectin Blotting

Unless stated otherwise, all the chemicals were purchased from Sigma–Aldrich (Poznań, Poland). Eight micrograms of seminal plasma protein, determined with Bradford [37] method, was loaded on the SDS-PAGE [38] gel lane for protein detection and 10 µg for Western blotting and lectin probing. The two gels were electrophoresed simultaneously, one of them stained with Sypro Ruby according to the manufacturer’s protocol [39], and the other transferred onto nitrocellulose membrane according to [40], 90 min at 120 mA. Each Sypro Ruby stained gel contained a lane loaded with 0.1 µg of bovine serum albumin (BSA), used for calculation of protein content in each particular protein band. Gels were acquired with a GBox F2 CCD camera (Syngene, Cambridge, UK) in transmitted UV light.

### 4.3. Lectin Reactivity

*Galanthus nivalis* lectin was applied to detect mannose-presenting glycans in SP glycoproteins. Like the other *Amaryllidaceae* lectins, GNL is mannose but not glucose specific. Moreover, the lectin does not bind glycoproteins with mannose residues hidden inside the structure of elongated, complex type glycans [41,42,43]. Yeast carboxypeptidase Y (CPY, 1 µg/gel lane) was used to standardize lectin staining. Nitrocellulose membranes after overnight blocking with 1% Tween-20 were incubated with biotinylated *Galanthus nivalis* lectin (GNL, Vector Labs, Peterborough, UK), diluted 1:500 in 15 mM Tris–HCl buffer, pH 7.4, with 0.15 M NaCl and 0.1% Tween-20 (TBST) for 1 h at room temperature (RT), with constant gentle shaking. The glycoprotein-lectin complexes were detected with ExtrAvidin-alkaline phosphatase (diluted 1:10,000 in TBST, 45 min, RT). After extensive washing (4 × 3 min), the membranes were submerged in DuoLux solution (Vector Labs, Peterborough, UK) for 5 min in the dark, constantly shaking, and washed three times with 0.1 M Tris-HCl buffer, pH 9.5, with 50 mM MgCl_2_. A GBox F2 CCD camera (Syngene, Cambridge, UK) with EPI-UV light was used for the acquisition of blots.

### 4.4. Densitometry

GeneTools 4.03 (Syngene, Cambridge, UK) software was used for the quantitative analysis of gels and blots. Lectin reactivity was expressed in arbitrary units, and one AU was defined as the GNL reactivity equal to that expressed by 1 µg of CPY. Fluorescent DuoLux staining provides linearity of signal in a wide range of analyte concentrations; thus errors resulting from band saturation can be avoided. Also, fluorescent protein staining maintains the linearity of signal versus protein concentration, so the protein content in each particular band was estimated as the ratio of sample/BSA band signals. The estimation of protein content in gel bands was used to calculate the density of mannose glycoepitopes, defined as the ratio of GNL reactivity to protein content [23,44,45].

### 4.5. Reactivity with Antibodies

Blots were incubated with polyclonal rabbit anti-PSA antibodies (Abcam, Cambridge, UK), diluted 1:200 in TBST for 1 h at RT, with constant gentle shaking, and next with horseradish peroxidase labeled anti-rabbit IgG (Jackson Antibodies, Suffolk, UK). For HRP staining diaminobenzidine (0.1 mg/mL) and H_2_O_2_ (0.04%) in 0.1 M citric buffer, pH 5.5, were applied.

### 4.6. Affinity Chromatography

GNL-reactive glycoproteins were isolated in affinity chromatography on agarose immobilized lectin (Vector Labs, Peterborough, UK). A seminal plasma bulk sample was prepared by pooling the equal protein amount of 30 infertile subjects. Five milligrams of protein was mixed with 2 mL of GNL-agarose in 0.05 M phosphate buffer, pH = 6.5, and stirred gently for 2 h at RT. The unbound proteins were washed out on the column and GNL-reactive glycoproteins were eluted with 0.5 M α-methyl-d-mannopyranoside in 0.1 M acetate buffer, pH = 4.0. The released proteins were immediately neutralized, dialyzed overnight to 0.1 M bicarbonate buffer and concentrated on viva-spin filters (MWCO 5 kDa, Sartorius Poland, Kostrzyn Wlkp, Poland, centrifuged for 20 min with 15,000× *g*).

### 4.7. Mass Spectrometry Identification of Proteins

Mass spectrometric identification of the proteins was performed in the Laboratory of Mass Spectrometry, Institute of Biochemistry and Biophysics, Polish Academy of Sciences, Warsaw, Poland.

Trypsin digestion was performed with the standard Shevchenko procedure [46] in the Coomassie Brilliant Blue stained bands excised from the gel (0.5% CBB in 40% methanol and 10% acetic acid). For in-solution trypsin digestion the protein sample, deglycosylated as described in the next paragraph, was reduced with dithiothreitol (10 mM) in 100 mM NH_4_HCO_3_ (30 min, 57 °C), and alkylated with 50 mM iodoacetamide (IAA, 45 min in the dark, RT). Trypsin was added in the enzyme:protein ratio 10:1, then the sample was digested overnight at 37 °C, and next acidified with 5% trifluoroacetic acid (TFA). Mass spectrometric analysis was done with a Q Exactive Focus Hybrid Quadrupole-Orbitrap Mass Spectrometer (Thermo Fisher Scientific, Waltham, MA, USA) and searched with the Mascot engine through the Swiss Prot database. Trypsin was specified as the proteolytic enzyme and 1 missing cleavage was allowed. Carbamidomethylation of cysteine was set as fixed modification and methionine oxidation was set as the only variable modification. Charge state of +1 was considered for parent ions. Tolerance of ±30 ppm was set for peptide and ±0.1 Da for MS/MS fragment mass accuracy. Only peptides identified with a significant Mascot score (*p* < 0.05) were considered.

### 4.8. Glycan Analysis by MALDI-TOF-MS

*N*-glycans were isolated according to Morelle and Michalski [47]. 100 ug of GNL-agarose purified material was incubated with 4% SDS and 4% βME in 50 mM NH_4_HCO_3_ (20 min, 100 °C), and next with 10% Nonidet P40 (5 min, RT). Glycans were cut off with 9 µL of PNGase F (4.5 mU, New England Biolabs, Hitchin, UK) through overnight incubation at 37 °C. Next, the protein was precipitated with cold ethanol (1:3 *v*/*v*), left for 2 h at −20 °C and centrifuged for 10 min at 8000× *g*. Supernatant containing glycans was lyophilized, dissolved in dimethyl superoxide, permethylated and further purified on a Sep-Pak C18 column (Thermo Fisher Scientific, Waltham, MA, USA).

Glycans were characterized by matrix-assisted laser desorption/ionization-time of flight mass spectrometry (MALDI-TOF-MS). All samples were prepared by mixing 1 microliter of glycan solution with 1 microliter of matrix solution. An initial solution of the DHB matrix was prepared by dissolving 20 mg of 2,5-dihydroxy benzoic acid (DHB) in 1 mL of 1:1, acetonitrile (ACN)/water. 1 microliter of glycan/matrix mixture was spotted onto the steel plate. Dried droplets were analyzed with a Bruker Ultraflex Extreme MALDI TOF/TOF mass spectrometer (Bruker Daltonics, Bremen, Germany) in reflection-positive ion mode. The samples were irradiated by a 2 kHz solid-state laser beam with the intensity to give the best signal-to-noise ratio for each sample. All spectra were obtained by accumulating 500 laser shots for quantification. The delayed extraction time and the accelerating voltage were adjusted to 100 ns and 25 kV, respectively. The ions were recorded in the range of 1500–4500 *m*/*z*. Calibration of the MALDI-TOF mass spectrometer was performed with the peptide calibration standard from Bruker Daltonics. All spectra were acquired by flexControl and then evaluated by flexAnalysis software (Bruker Daltonics, Bremen, Germany). Glycan structures were identified with GlycoWorkbench 1.1.3480 software [26,27].

### 4.9. Statistical Analysis

Statistica 10.0 software (StatSoft Poland, Kraków, Poland) was used for basic statistics. The Kruskal–Wallis test was applied to verify statistical significance of differences among the subject groups, and *p* values <0.05 were considered significant.

## References

[1] Wassarman P.M. (1995). Towards molecular mechanisms for gamete adhesion and fusion during mammalian fertilization. Curr. Opin. Cell Biol..

[2] Seppälä M., Koistinen H., Koistinen R., Chiu P.C., Yeung W.S. (2007). Glycosylation related actions of glycodelin: Gamete, cumulus cell, immune cell and clinical associations. Hum. Reprod. Update.

[3] Pang P.C., Chiu P.C., Lee C.L., Chang L.Y., Panico M., Morris H.R., Haslam S.M., Khoo K.H., Clark G.F., Yeung W.S. (2011). Human sperm binding is mediated by the sialyl-Lewis(x) oligosaccharide on the zona pellucida. Science.

[4] Alavi A., Axford J.S. (2008). Glyco-biomarkers: Potential determinants of cellular physiology and pathology. Dis. Markers.

[5] Alavi A., Axford J.S. (2008). Sweet and sour: The impact of sugars on disease. Rheumatology.

[6] Pang P.C., Tissot B., Drobnis E.Z., Morris H.R., Dell A., Clark G.F. (2009). Analysis of the human seminal plasma glycome reveals the presence of immunomodulatory carbohydrate functional groups. J. Proteome Res..

[7] White K.Y., Rodemich L., Nyalwidhe J.O., Comunale M.A., Clements M.A., Lance R.S., Schellhammer P.F., Mehta A.S., Semmes O.J., Drake R.R. (2009). Glycomic characterization of prostate-specific antigen and prostatic acid phosphatase in prostate cancer and benign disease seminal plasma fluids. J. Proteome Res..

[8] Stanley P., Schachter H., Taniguchi N., Varki A., Cummings R.D., Esko J.D., Freeze H.H., Stanley P., Bertozzi C.R., Hart G.W., Etzler M.E. (2009). *N*-Glycans. Essentials of Glycobiology.

[9] Morris H.R., Dell A., Easton R.L., Panico M., Koistinen H., Koistinen R., Oehninger S., Patankar M.S., Seppala M., Clark G.F. (1996). Gender-specific glycosylation of human glycodelin affects its contraceptive activity. J. Biol. Chem..

[10] Yeung W.S., Lee K.F., Koistinen R., Koistinen H., Seppala M., Ho P.C., Chiu P.C. (2006). Roles of glycodelin in modulating sperm function. Mol. Cell. Endocrinol..

[11] Yeung W.S., Lee K.F., Koistinen R., Koistinen H., Seppälä M., Chiu P.C. (2009). Effects of glycodelins on functional competence of spermatozoa. J. Reprod. Immunol..

[12] Mattsson J.M., Valmu L., Laakkonen P., Stenman U.H., Koistinen H. (2008). Structural characterization and anti-angiogenic properties of prostate-specific antigen isoforms in seminal fluid. Prostate.

[13] Fukushima K., Satoh T., Baba S., Yamashita K. (2010). Alpha1,2-Fucosylated and beta-*N*-acetyl-galactosaminylated prostate-specific antigen as an efficient marker of prostatic cancer. Glycobiology.

[14] Lefièvre L., Bedu-Addo K., Conner S.J., Machado-Oliveira G.S., Chen Y., Kirkman-Brown J.C., Afnan M.A., Publicover S.J., Ford W.C., Barratt C.L. (2007). Counting sperm does not add up any more: Time for a new equation?. Reproduction.

[15] Barratt C.L., Mansell S., Beaton C., Tardif S., Oxenham S.K. (2011). Diagnostic tools in male infertility—The question of sperm dysfunction. Asian J. Androl..

[16] Esteves S.C., Miyaoka R., Agarwal A. (2011). An update on the clinical assessment of the infertile male. Clinics.

[17] Hwang K., Lipshultz L.I., Lamb D.J. (2011). Use of diagnostic testing to detect infertility. Curr. Urol. Rep..

[18] Franken D.R., Oehninger S. (2012). Semen analysis and sperm function testing. Asian J. Androl..

[19] Nagler H.M. (2011). Male factor infertility: A solitary semen analysis can never predict normal fertility. Nat. Rev. Urol..

[20] Kratz E.M., Faundez R., Katnik-Prastowska I. (2011). Fucose and sialic acid expressions in human seminal fibronectin and α_1_-acid glycoprotein associated with leukocytospermia of infertile men. Dis. Markers.

[21] Kratz E.M., Ferens-Sieczkowska M., Faundez R., Kątnik-Prastowska I. (2014). Changes in fucosylation of human seminal IgG and secretory component of IgA in leukocytospermic patients. Glycoconj. J..

[22] Kratz E.M., Ferens-Sieczkowska M. (2014). Association of IgA secretory component sialylation with leucocytospermia of infertile men—A pilot study. Andrologia.

[23] Olejnik B., Kratz E.M., Zimmer M., Ferens-Sieczkowska M. (2015). Glycoprotein fucosylation is increased in seminal plasma of subfertile men. Asian J. Androl..

[24] Ceroni A., Maass K., Geyer H., Geyer R., Dell A., Haslam S.M. (2008). GlycoWorkbench: A tool for the computer-assisted annotation of mass spectra of glycans. J. Proteome Res..

[25] Damerell D., Ceroni A., Maass K., Ranzinger R., Dell A., Haslam S.M. (2012). The GlycanBuilder and GlycoWorkbench glycoinformatics tools: Updates and new developments. Biol. Chem..

[26] Juyena N.S., Stelletta C. (2012). Seminal plasma: An essential attribute to spermatozoa. J. Androl..

[27] Bromfield J.J. (2014). Seminal fluid and reproduction: Much more than previously thought. J. Assist. Reprod. Genet..

[28] Bedford J.M. (2015). The functions—Or not—Of seminal plasma?. Biol. Reprod..

[29] Rickard J.P., Pini T., Soleilhavoup C., Cognie J., Bathgate R., Lynch G.W., Evans G., Maxwell W.M., Druart X., de Graaf S.P. (2014). Seminal plasma aids the survival and cervical transit of epididymal ram spermatozoa. Reproduction.

[30] Leahy T., de Graaf S.P. (2012). Seminal plasma and its effect on ruminant spermatozoa during processing. Reprod. Domest. Anim..

[31] Rickard J.P., Schmidt R.E., Maddison J.W., Bathgate R., Lynch G.W., Druart X., de Graaf S.P. (2014). Variation in seminal plasma alters the ability of ram spermatozoa to survive cryopreservation. Reprod. Fertil. Dev..

[32] Rodríguez-Martínez H., Kvist U., Ernerudh J., Sanz L., Calvete J.J. (2011). Seminal plasma proteins: What role do they play?. Am. J. Reprod. Immunol..

[33] Druart X., Rickard J.P., Mactier S., Kohnke P.L., Kershaw-Young C.M., Bathgate R., Gibb Z., Crossett B., Tsikis G., Labas V. (2013). Proteomic characterization and cross species comparison of mammalian seminal plasma. J. Proteomics.

[34] Varki A., Freeze H.F., Gagneux P., Varki A., Cummings R.D., Esko J.D., Freeze H.H., Stanley P., Bertozzi C.R., Hart G.W., Etzler M.E. (2009). Evolution of glycan diversity. Essentials of Glycobiology.

[35] Chiu P.C., Chung M.K., Tsang H.Y., Koistinen R., Koistinen. H., Seppala M., Lee K.F., Yeung W.S. (2005). Glycodelin-S in human seminal plasma reduces cholesterol efflux and inhibits capacitation of spermatozoa. J. Biol. Chem..

[36] World Health Organization (2010). WHO Laboratory Manual for the Examination and Processing of Human Semen.

[37] Bradford M.M. (1976). A rapid and sensitive method for the quantitation of microgram quantities of protein utilizing the principle of protein-dye binding. Anal. Biochem..

[38] Laemmli U.K. (1970). Cleavage of structural proteins during the assembly of the head of bacteriophage T4. Nature.

[39] Sigma Aldrich Co. SYPRO^®^Ruby Protein Gel Stain. Product S 4942 Datasheet.

[40] Towbin H., Stachelin T., Gordon J. (1979). Electrophoretic transfer of proteins from polyacrylamide gels to nitrocellulose sheets: Procedure and some applications. Proc. Natl. Acad. Sci. USA.

[41] Shibuya N., Goldstein I.J., van Damme E.J., Peumans W.J. (1988). Binding properties of a mannose-specific lectin from the snowdrop (*Galanthus nivalis*) bulb. J. Biol. Chem..

[42] Barre A., Bourne Y., van Damme E.J., Peumans W.J., Rougé P. (2001). Mannose-binding plant lectins: Different structural scaffolds for a common sugar-recognition process. Biochimie.

[43] Van Damme E.J., Nakamura-Tsuruta S., Smith D.F., Ongenaert M., Winter H.C., Rougé P., Goldstein I.J., Mo H., Kominami J., Culerrier R. (2007). Phylogenetic and specificity studies of two-domain GNA-related lectins: Generation of multispecificity through domain duplication and divergent evolution. Biochem. J..

[44] Kossowska B., Ferens-Sieczkowska M., Gancarz R., Passowicz-Muszyńska E., Jankowska R. (2005). Fucosylation of serum glycoproteins in lung cancer patients. Clin. Chem. Lab. Med..

[45] Ferens-Sieczkowska M., Kossowska B., Gancarz R., Dudzik D., Knas M., Popko J., Zwierz K. (2007). Fucosylation in synovial fluid as a novel clinical marker for differentiating joint diseases—A preliminary study. Clin. Exp. Rheumatol..

[46] Shevchenko A., Tomas H., Havlis J., Olsen J.V., Mann M. (2006). In-gel digestion for mass spectrometric characterization of proteins and proteomes. Nat. Protoc..

[47] Morelle W., Michalski J.C. (2007). Analysis of protein glycosylation by mass spectrometry. Nat. Protoc..

